# Trajectories of Food Choice Motives and Weight Status of Malaysian Youths during the COVID-19 Pandemic

**DOI:** 10.3390/nu13113752

**Published:** 2021-10-23

**Authors:** Seok Tyug Tan, Chin Xuan Tan, Seok Shin Tan

**Affiliations:** 1 Department of Healthcare Professional, Faculty of Health and Life Sciences, Management and Science University, University Drive, Off Persiaran Olahraga, Seksyen 13, Shah Alam 40100, Malaysia; sttan@msu.edu.my; 2 Department of Allied Health Sciences, Faculty of Science, Universiti Tunku Abdul Rahman, Jalan Universiti, Bandar Barat, Kampar 31900, Malaysia; tancx@utar.edu.my; 3 Department of Nutrition and Dietetics, School of Health Sciences, International Medical University, Bukit Jalil, Kuala Lumpur 57000, Malaysia

**Keywords:** food choice motives, weight status, youths, COVID-19, lockdown

## Abstract

Stay-at-home orders have abruptly altered food purchasing behaviour, dietary habits, and food choice motives. Therefore, this study aims to investigate the trajectory of food choice motives and their associations with the weight status of Malaysian youths in the time of COVID-19. Socio-demographic information and anthropometric measurements were self-reported by the respondents, while the food choice motives were assessed using a validated 38-item food choice questionnaire (FCQ). Of the 1013 Malaysian youths, 48.6% gained weight due to the confinement, with an average weight gain of 3.90 ± 2.92 kg. On the other hand, 47.0% to 73.0% of the youths changed their food choice motives in the time of COVID-19. Of the 10 motives, convenience (48.5%) had the largest percentage increase, followed by weight control (47.0%) and health (45.3%). Moreover, the mean scores of health (*t* = −3.324, *p* = 0.001), convenience (*t* = −5.869, *p* < 0.001), weight control (*t* = −7.532, *p* < 0.001), natural content (*t* = −5.957, *p* < 0.001), ethical concern (*t* = −4.419, *p* < 0.001) and price (*t* = −3.737, *p* < 0.001) were significantly higher during the pandemic compared to pre-pandemic. Findings from the multinomial regression model revealed that youths highly concerned for weight control were more likely to be in the weight loss category (Adjusted odds ratio (AOR) = 1.633, Confidence Interval (CI) = 1.230–2.168, *p* = 0.001). Conversely, those who gained weight due to the pandemic confinement highly valued natural content in foods (AOR = 0.653, CI = 0.481–0.886, *p* = 0.006) when making their food choices in this unprecedented pandemic. In conclusion, Malaysian youths made healthier food choices to mitigate the risk of COVID-19 infection.

## 1. Introduction

In Malaysia, confirmed COVID-19 cases continue to rise despite the implementation of three nationwide lockdowns (commonly known as Movement Control Orders, MCOs). As of September 2021, more than 1.8 million Malaysians were infected by this highly contagious virus [1]. The reported cases have already accounted for approximately 5.5% of the 32.7 million population in Malaysia [2]. In line with the enforcement of MCOs, several stringent measures were taken by the Federal Government of Malaysian to curb the spread of COVID-19 in communities. Some of those preventive measures included the temporary closure of non-essential industries and learning institutions, only two in a family being permitted to go grocery shopping, and an imposed a travel limit of no more than 10 km radius from an individual’s residential address [3].

The stay-at-home orders abruptly altered food purchasing behaviour, dietary habits, and food choice motives [4,5,6]. A recent report released by the Department of Statistics Malaysia [7] indicated that the e-commerce income for the first quarter of 2021 surged 30% year-on-year. Alternatively, it could simply mean that the COVID-19 crisis increased e-commerce and digital marketing in Malaysia due to the fears of infection. The shift from physical store visits to online shopping for necessities, in addition to the enforcement of nationwide lockdowns, may have led to a change in Malaysians’ dietary habits and food choice motives. A study by Abdullah, Mersat, and Wong [8] stated that Malaysians reduced grocery trips to wet markets and supermarkets during the implementation of MCO as the result of travel restrictions. Limited access to wet markets during the lockdown appears to be the cause of the poor availability of fresh produce such as fruits, vegetables, fish, and flesh meats at the household level [9]. To the best of the authors’ knowledge, there is no prior study examining the food choice motives of Malaysians during COVID-19. Therefore, this study aims to investigate the trajectory of food choice motives and their associations with the weight status of Malaysian youths throughout the COVID-19 pandemic.

## 2. Methodology

### 2.1. Study Design and Population

Data collection for this cross-sectional study was conducted from 4–11 June 2021 (during the re-enforcement of MCO) using a combination of convenience and snowball sampling approaches. Malaysian youths aged 18–30 from all ethnicities (Malay, Chinese, Indian, indigenous people of Sabah and Sarawak), with access to internet during the pandemic, and the ability to read English were included in this study. The current study was conducted in accordance with the Declaration of Helsinki, in which ethical approval was obtained from the Research Ethics Committee of Management and Science University, Malaysia (Reference number: MSU-RMC-02/FR01/06/L1/040). A web-based anonymous self-administrated questionnaire was hosted on google forms and circulated to prospective respondents through social media platforms including WhatsApp, Facebook, Twitter, Instagram, and TikTok. Informed consent was obtained from the respondents before revealing the first survey question of this study. The sample size was computed based on Krejcie and Morgan [10], assuming a 5% margin of error, 99% confidence level, and 9 million Malaysian youths as of 2020 [11]. Thus, the minimum sample size required for this study was 663 respondents. A total of 1055 respondents answered this survey; however, findings in the current study were tabulated based on the responses from 1013 Malaysian youths, after excluding those who were not the correct age, provided duplicate responses, or were non-Malaysian.

### 2.2. Socio-Demographic Characteristics and Self-Reported Weight Trajectory

Socio-demographic information, including gender, age, ethnicity, and marital status was self-reported by the respondents. To determine their weight trajectory status during COVID-19, the respondents were instructed to recall body weight before the pandemic (kg) and measure their current body weight (kg) using a bathroom scale at home whenever possible. Weight trajectory was deemed the difference between current and pre-pandemic body weights [12]. All respondents were also required to report their body height (cm) for the estimation of body mass index (BMI) (kg/m^2^). The BMI was then stratified into 4 categories (underweight, normal, overweight, and obese) in accordance with the Asia-Pacific cut-off points [13].

### 2.3. Assessment of Food Choice Motives

Food choice motives before and during the pandemic were assessed with a validated 38-item food choice questionnaire (FCQ) [14]. This 38-item was further sorted into 10 factors: convenience (5-item), weight control (3-item), health (6-item), natural content (3-item), mood (6-item), ethical concern (3-item), price (3-item), familiarity (3-item), sensory appeal (4-item), and religion (2-item). Each item was rated with a 5-point Likert scale ranging from “not very important” (1 point) to “very important” (5 points). The respondents were required to answer two sets of FCQs, which were intended to assess their food choice motives before the pandemic (38-item) and the current food choice motives (food choice motives during the pandemic) (38-item). The mean score was calculated by averaging the total score to the number of items in a factor. Trajectory in the food choice motives (∆food choice motive) was reflected by the difference in the food choice motives before and during the pandemic.

### 2.4. Statistical Analysis

Data analysis was carried out using IBM SPSS statistics 26.0 (IBM Corp., Armonk, NY, USA). Exploratory factor analysis (EFA) and Cronbach’s alpha were used to measure the internal consistency and reliability of all items of the FCQ, respectively. Frequency, percentage, mean, and standard deviation (SD) were used to describe variables where appropriate. All continuous variables were considered as normally distributed if the skewness was ±2. Trajectories in the food choice motives and weight status before and during the COVID-19 pandemic were tested with a paired-samples *t*-test. To identify the food choice motives that contributed to weight trajectory during the pandemic, a univariate analysis (one-way ANOVA) was performed to determine the mean difference between groups (weight loss, sustained weight, and weight gain). Food choice motives (independent variables) which portrayed a *p*-value of less than 0.25 in one-way ANOVA analysis were further analysed with multinomial logistic regression (multivariable analysis) [15]. Multicollinearity was tested to ensure the independent variables included in the regression model did not violate the assumption. Findings of the multinomial regression were presented as adjusted odds ratio (AOR) with a 95% confidence interval (CI). A *p*-value of less than 0.05 (*p* < 0.05) was statistically significant.

## 3. Results

Table 1 indicates the socio-demographic characteristics and weight status of the respondents. Of the 1013 respondents, the majority were female (64.4%), aged 18–24 (80.7%), Malay (59.4%), and with single marital status (94.8%). Emerging findings demonstrated that 48.6% of the youths gained weight due to the confinement, with an average weight gain of 3.90 ± 2.92 kg. Conversely, 36.5% of the youths declared a lighter body weight during the pandemic than pre-pandemic, with an average weight loss of 4.70 ± 3.99 kg. There was no significant difference in the BMI of the youths throughout the pandemic (pre-pandemic BMI: 22.72 ± 4.82 kg/m^2^ versus BMI during the pandemic: 22.78 ± 4.68 kg/m^2^) (*t* = −1.129, *p* = 0.259); however, a marginal increase in the prevalence of overweight/obesity was observed (pre-pandemic: 39.5% versus during the pandemic: 40.6%).

Table 2 demonstrates the internal consistency and reliability of all items of the FCQ. Since items with factor loading greater than 0.4 and Cronbach’s alpha above 0.7 are good representations of a factor [16], no item was discarded in the subsequent data analysis.

**Figure 1:**
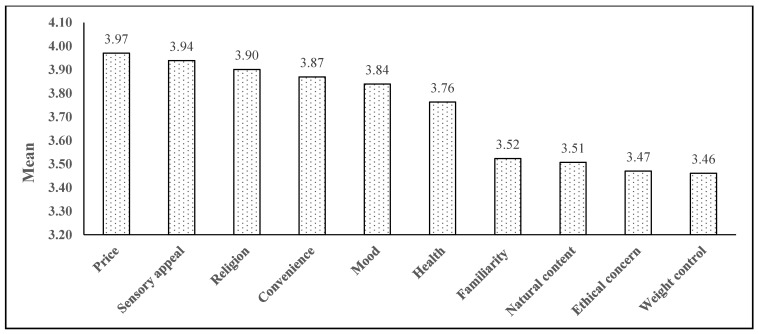
Ranking of food choice motives before the COVID-19 pandemic.

**Figure 2:**
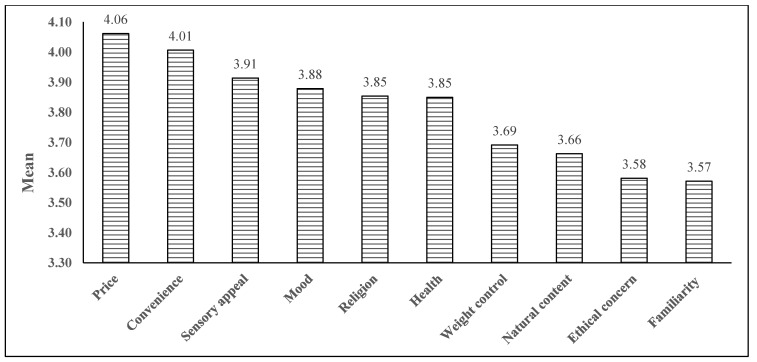
Ranking of food choice motives during the COVID-19 pandemic.

Table 3 shows the trajectory of food choice motives before and during the COVID-19 pandemic. This study revealed that 47.0% (religion) to 73.0% (health) of the youths changed their food choice motives due to the pandemic lockdown. Of the 10 motives, convenience (48.5%) had the largest percentage increase, followed by weight control (47.0%) and health (45.3%). Moreover, the mean scores of health (*t* = −3.324, *p =* 0.001), convenience (*t* = −5.869, *p <* 0.001), weight control (*t* = −7.532, *p <* 0.001), natural content (*t* = −5.957, *p <* 0.001), ethical concern (*t* = −4.419, *p <* 0.001), and price (*t* = −3.737, *p <* 0.001) were significantly higher during the pandemic compared to pre-pandemic. In addition, the food choice motives before and during the pandemic were also ranked by mean scores (Figure 1 and Figure 2). Before the COVID-19 pandemic, price (3.97 ± 0.88), sensory appeal (3.94 ± 0.82), and religion (3.90 ± 1.06) were the three top-rated food choice motives of Malaysian youths. After 15 months of the COVID-19 outbreak in Malaysia, price remained the motive with the highest mean score (4.06 ± 0.99), followed by convenience (4.01 ± 0.93), and sensory appeal (3.91 ± 0.94).

Table 4 depicts the mean difference in food choice motives before and during the pandemic by weight status. There was no significant difference (*p* > 0.05) in the mean scores of all food choice motives by weight status before the pandemic outbreak in Malaysia. Paradoxically, the mean score of health (*F* = 6.505, *p* = 0.002), convenience (*F* = 5.405, *p* = 0.005), weight control (*F* = 9.384, *p* < 0.001), and mood (*F* = 3.479, *p* = 0.031) significantly differed by weight status during the COVID-19 pandemic. Food choice motives that portrayed a *p*-value of less than 0.25 (*p* < 0.25) in one-way ANOVA analysis were selected as the predictors for weight trajectory in multinomial regression. Thus, health, convenience, weight control, mood, natural content, sensory appeal, and price were included in the regression model (Table 5). Findings presented in the regression model confirmed that weight control and natural content were the significant predictors for weight trajectory during the pandemic lockdown. Youths who were highly concerned for weight control were more likely in the weight loss category (AOR = 1.633, CI = 1.230–2.168, *p* = 0.001). Conversely, those who gained weight due to the pandemic confinement highly valued natural content in foods (AOR = 0.653, CI = 0.481–0.886, *p* = 0.006) when making their food choices in this unprecedented pandemic.

## 4. Discussion

Emerging findings revealed that the prevalence of overweightness/obesity increased from 39.5% (pre-pandemic) to 40.6% (during the pandemic) due to the pandemic lockdown. Furthermore, 48.6% of the youths gained an average body weight of 3.90 ± 2.92 kg after 15 months of the COVID-19 outbreak in Malaysia. A similar trend of findings was obtained in Tan, Tan, and Tan [5], whereby it is reported that Malaysian university students who gained weight in the first 4 months of pandemic confinement put on an additional 3.9 kg. Malaysian youths in the current study had a higher percentage of weight gain than adults in Belgium (28.6% in April 2020) [17], adults in China (30.6% in March/April 2020) [18] and adults in Sri Lanka (38.5% in May/June 2021) [19].

An appreciable proportion of Malaysian youths (47.0% to 73.0%) altered their food choice motives due to the pandemic. Overall these findings are in accordance with a recent study by Marty, de Lauzon-Guillain, Labesse, and Nicklaus [6] among adults residing in France during the COVID-19 lockdown. Interestingly, it was observed that price was rated with the highest mean scores regardless of the pandemic situation. Convenience surpassed sensory appeal and ranked as the second highest-rated food choice motive in pandemic confinement. The COVID-19 economic recession drove a spike in the unemployment rate and caused devastating losses in incomes. Youths, who generally have the least work experience, have been more likely to drift out of the labour market in this unprecedented crisis [20]. Changes in the income elasticity of food demand during the COVID-19 pandemic may partly explain why food price emerged as the most important determinant of food choice among youths in Malaysia.

Of the 10 food choice motives, no significant difference in the mean score of mood, familiarity, sensory appeal, and religion was observed. Food decisions in accordance with these previously mentioned motives were remarkably resilient despite the COVID-19 outbreak. Moreover, familiarity was also rated a relatively low priority during the pandemic lockdown. These findings were broadly in line with previous studies by Asma et al. [14] and Mohd-Any, Mahdzan, and Cher [21], which reported that familiarity was the least prominent food choice motive among Malaysian adults. Another important finding to be highlighted is that mood was not the main determinant for food choice, although there are a few studies that have demonstrated that the pandemic lockdown was associated with increased emotional eating and binge eating [22,23,24].

The pandemic lockdown posed a profound impact on the food choice motives of Malaysian youths. Food decisions during the COVID-19 lockdown relied heavily on convenience (a rise of 48.5%), weight control (a rise of 47.0%), health (a rise of 45.3%), and natural content (a rise of 44.1%), signalling that Malaysian youths made healthier food choices compared to previously (Table 3). However, these findings were unsurprising, because obesity is recognized as one of the predisposing factors for COVID-19 [25]. People may deliberately demand nutritious foods or functional foods with reasonable health claims to reduce the risk of infection. Fear of infection, engagement in healthy eating as a means of obtaining optimum immunity, and a greater health consciousness during the pandemic could be among the factors that drove healthier food choices [26]. On the other hand, convenience recorded the highest percentage increase among the 10 food choice motives. The increase was mainly driven by more home cooking and travel restrictions enacted by the Federal Government of Malaysian during the nationwide lockdowns [27].

It has been previously reported that unhealthy food choices contribute to obesity and its relevant co-comorbidities, including hypertension, type II diabetes, and cardiovascular diseases [28]. In line with those previously mentioned, the current study also examined the association between food choice motives and weight trajectories over the pandemic confinement. Of the seven motives in the multinomial regression model, only weight control and natural content were associated with the weight trajectory status. Youths who took weight control into consideration when making food choices were more likely to lose weight during the pandemic confinement (AOR = 1.633, CI = 1.230–2.168, *p* = 0.001). These findings generally support the notion that cutting back on calories and fat was an effective strategy for shedding weight during the COVID-19 lockdown [29]. On the contrary, youths who gained weight due to the pandemic lockdown showed a greater tendency of selecting foods with natural content than those in the weight loss category (AOR = 0.653, CI = 0.481–0.886, *p* = 0.006). One possible explanation is that some functional foods that come with natural content may also contain significant amounts of added sugars or salt [30]. Overconsumption of functional foods with added sugar can be the underlying reason for gaining additional weight in this unprecedented pandemic.

Findings presented in the current study must be interpreted within the context of limitations. Respondents in the current study were required to retrospectively recall their pre-pandemic food choice motives and body weight, which can potentially lead to reporting biases. Findings in the current study may not reflect the food choice motives of all Malaysian youths, which is attributable to the fact that those aged 18–24 and/or with single marital status were over-represented. In view of the enactment of physical distancing order, data collection was conducted using Google Forms, which requires internet access. This approach had ruled out those without internet access from taking part in this study. Despite those previously mentioned, this study is the first that investigated changes in the food choice motives and weight status of Malaysian youths in the time of COVID-19.

## 5. Conclusions

The COVID-19 pandemic has altered the food choice motives of Malaysian youths, in addition to a surge in the prevalence of overweightness/obesity. Health, convenience, weight control, natural content, ethical concern, and price were important drivers of food choice during the pandemic lockdown. In general, youths in the current study made healthier food choices to mitigate the risk of COVID-19 infection. Understanding changes in food choice motives may be useful in the formulation of relevant intervention strategies to ease the burden of overweightness/obesity among Malaysian youths in the post COVID-19 era. In addition, interventions targeting those who gained weight during the pandemic should focus on food label reading for the selection of wiser functional food choices in future.

## Figures and Tables

**Table 1:** Socio-demographic characteristics and weight status of the respondents.

Variable	Frequency, *n* (%)	Mean ± Standard Deviation
Gender		
Male	361 (35.6)	-
Female	652 (64.4)	
Age (years old)		
18–24	817 (80.7)	
25–30	196 (19.3)	22.65 ± 2.46
Ethnicity		
Malay Chinese Indian Others (Indigenous people of Sabah and Sarawak)	602 (59.4) 164 (16.2) 226 (22.3) 21 (2.1)	-
Marital Status		
Single Married Divorced	960 (94.8) 49 (4.8) 4 (0.4)	-
Weight status (kg)		
Sustained weight	151 (14.9)	-
Weight loss Weight gain	370 (36.5) 492 (48.6)	−4.70 ± 3.99 3.90 ± 2.92
BMI status (kg/m^2^) ^1^		
Before the pandemic		
Underweight Normal Overweight Obese	180 (17.8) 433 (42.7) 157 (15.5) 243 (24.0)	22.72 ± 4.82 ^a^
During the pandemic		
Underweight Normal Overweight Obese	158 (15.6) 444 (43.8) 160 (15.8) 251 (24.8)	22.78 ± 4.68 ^a^

^1^ Mean difference was analysed with paired samples *t*-test. A different letter indicates significant difference at *p* < 0.05.

**Table 2:** Factor loading and Cronbach’s alpha of food choice questionnaire.

Food Choice Motives	Item	Before the Pandemic		During the Pandemic	
		Loading	Cronbach’s Alpha	Loading	Cronbach’s Alpha
Health	Is high in fibre	0.824	0.935	0.896	0.954
	Is nutritious	0.884		0.923	
	Contains a lot of vitamins and minerals	0.895		0.913	
	Is high in protein	0.864		0.885	
	Keeps me healthy	0.882		0.915	
	Is good for my skin/teeth/hair/nails, etc.	0.863		0.875	
Convenience	Is easy to prepare	0.750	0.858	0.829	0.916
	Is easily available in shops/supermarkets	0.823		0.871	
	Can be cooked very simply	0.842		0.906	
	Takes no time to prepare	0.786		0.860	
	Can be bought in shops close to where I live/work	0.799		0.866	
Weight control	Is low in calories	0.883	0.857	0.935	0.919
	Is low in fat	0.898		0.938	
	Helps me control my weight	0.866		0.910	
Mood	Cheers me up	0.804	0.917	0.885	0.946
	Helps me cope with stress	0.881		0.904	
	Keeps me awake	0.759		0.819	
	Helps me relax	0.894		0.926	
	Makes me feel good	0.864		0.892	
	Helps me cope with life	0.854		0.903	
Natural content	Contains no additives	0.855	0.833	0.914	0.905
	Contains natural ingredients	0.875		0.918	
	Contains no artificial ingredients	0.868		0.919	
Familiarity	Is familiar	0.821	0.784	0.868	0.852
	Is like the food I ate when I was a child	0.813		0.879	
	Is what I usually eat	0.877		0.892	
Sensory appeal	Tastes good	0.788	0.843	0.872	0.908
	Smells nice	0.881		0.902	
	Has a pleasant structure	0.797		0.881	
	Looks nice	0.838		0.890	
Ethical concern	Is packaged in an environmentally friendly way	0.849	0.820	0.873	0.863
	Comes from the country I approve of politically	0.863		0.894	
	Has the country of origin clearly marked	0.862		0.893	
Price	Is not expensive	0.900	0.842	0.926	0.897
	Is cheap	0.911		0.932	
	Is good value for money	0.801		0.872	
Religion	Has certification from the government	0.903	0.772	0.921	0.820
	Permissible by religion	0.903		0.921	

**Table 3:** Trajectory of food choice motives before and during the COVID-19 pandemic.

Food Choice Motive	Before the COVID-19 (Mean ± SD)	During the COVID-19 (Mean ± SD)	*t*-Value (*p*-Value ^1^)	Trajectory of Food Choice Motive (%)			
				Decreased	Sustained	Increased	∆Food Choice Motive
Health	3.76 ± 0.90	3.85 ± 1.00	−3.324 (0.001) *	27.6	27.0	45.3	73.0
Convenience	3.87 ± 0.83	4.01 ± 0.93	−5.869 (<0.001) *	23.7	27.8	48.5	72.2
Weight control	3.46 ± 1.00	3.69 ± 1.10	−7.532 (<0.001) *	21.8	31.2	47.0	68.8
Mood	3.84 ± 0.88	3.88 ± 0.98	−1.748 (0.081)	27.5	31.9	40.6	68.1
Natural content	3.51 ± 0.95	3.66 ± 1.06	−5.957 (<0.001) *	22.8	33.1	44.1	66.9
Familiarity	3.52 ± 0.94	3.57 ± 1.04	−1.938 (0.053)	28.2	34.0	37.8	66.0
Sensory appeal	3.94 ± 0.82	3.91 ± 0.94	1.124 (0.263)	32.3	34.7	33.0	65.3
Ethical concern	3.47 ± 1.02	3.58 ± 1.08	−4.419 (<0.001) *	24.7	35.1	40.2	64.9
Price	3.97 ± 0.88	4.06 ± 0.99	−3.737 (<0.001) *	23.2	38.1	38.7	61.9
Religion	3.90 ± 1.06	3.85 ± 1.16	1.918 (0.055)	24.1	53.0	22.9	47.0

^1^ Mean difference was analysed with paired samples *t*-test. * Significant difference was considered at *p* < 0.05.

**Table 4:** Mean difference in food choice motives before and during the COVID-19 outbreak by weight status.

Food Choice Motive	Before the COVID-19		During the COVID-19	
	Weight Status (*F* Value) ^1^	*p*-Value ^2^	Weight Status (*F* Value) ^1^	*p*-Value ^2^
Health	0.009	0.991	6.505	0.002
Convenience	0.872	0.419	5.405	0.005
Weight control	1.053	0.349	9.384	<0.001
Mood	0.481	0.619	3.479	0.031
Natural content	0.325	0.723	2.746	0.065
Familiarity	0.844	0.430	1.042	0.353
Sensory appeal	0.542	0.582	2.611	0.074
Ethical concern	0.264	0.768	0.906	0.404
Price	0.626	0.535	2.759	0.064
Religion	0.144	0.866	0.605	0.547

^1^ Mean difference was tested with one-way ANOVA with Games–Howell post-hoc test. ^2^ Food choice motive that portrays a *p*-value of less than 0.25 (*p* < 0.25) was selected for multinomial logistic regression analysis.

**Table 5:** Multinomial logistic regression model of food choice motives during the COVID-19.

Food Choice Motive	Sustained Weight		Weight Loss	
	AOR (95% CI)	*p*-Value	AOR (95% CI)	*p*-Value
Health	1.139 (0.717–1.809)	0.582	1.291 (0.907–1.838)	0.156
Convenience	1.174 (0.751–1.836)	0.482	1.354 (0.967–1.895)	0.078
Weight control	1.176 (0.821–1.684)	0.377	1.633 (1.230–2.168)	0.001 *
Mood	0.833 (0.537–1.291)	0.414	0.920 (0.651–1.300)	0.637
Natural content	0.950 (0.632–1.426)	0.804	0.653 (0.481–0.886)	0.006 *
Sensory appeal	0.784 (0.501–1.227)	0.287	0.828 (0.586–1.169)	0.283
Price	1.075 (0.751–1.541)	0.692	0.902 (0.694–1.172)	0.441

Reference group: weight gain. * Significant difference was considered at *p* < 0.05.
